# Diagnostic Stewardship for Healthcare-Associated Infections: Opportunities and Challenges to Safely Reduce Test Use

**DOI:** 10.1017/ice.2017.278

**Published:** 2018-01-14

**Authors:** Gregory R. Madden, Robert A. Weinstein, Costi D. Sifri

**Affiliations:** 1. Division of Infectious Diseases and International Health, Department of Medicine, University of Virginia Health System, Charlottesville, Virginia; 2. Divisions of Infectious Diseases, Rush University Medical Center and Cook County Health and Hospitals System, both in Chicago, Illinois; 3. Office of Hospital Epidemiology/Infection Prevention & Control, University of Virginia Health System, Charlottesville, Virginia.

Healthcare-associated infections (HAIs) are associated with increased morbidity and mortality, prolonged hospital stays, and unnecessary cost. The financial stakes of HAIs for hospitals were underscored in 2008 when the Centers for Medicare and Medicaid Services (CMS) began to withhold payment for certain “reasonably preventable” HAIs, including catheter-associated urinary tract infections (CAUTIs), central line-associated bloodstream infections (CLABSIs), and surgical site infections (SSIs).^1^

Most current efforts to reduce HAIs focus on strategies to prevent infection without addressing unnecessary testing or diagnostic error; however, a false-positive test result that provides an erroneous diagnosis of an HAI may lead to increased cost and possible harm to the patient, although data quantifying these effects are lacking. Accurate diagnostics are critical for safe patient care and have additional impacts in our environment of value-based payment, public reporting, and quality metrics, where hospitals may incur penalties for HAI test overuse, including lost reimbursement, financial penalties, and damage to institutional reputation and rankings. From a patient care perspective, overdiagnosis of HAIs could lead to inappropriate antimicrobial use and attendant unnecessary cost and risks antimicrobial resistance and adverse drug effects.

## DIAGNOSTIC STEWARDSHIP CONCEPT AND ROLE IN HAIS

Developed more than 20 years ago, antimicrobial stewardship programs (ASPs) can play a key role in reducing cost, antimicrobial resistance, and some HAIs. Studies suggest that ASPs are most effective when coupled with infection prevention strategies.^2^ Overall, ASPs are widely adopted and regarded as safe and have not been found to increase patient mortality or other patient-centered adverse outcomes, despite reduced antimicrobial use.^3^ Recognizing this, The Joint Commission now requires ASPs for hospital accreditation, and the CMS has proposed ASP standards in acute-care hospitals, critical-access hospitals, and long-term care facilities.^4^

Diagnostic stewardship practices are increasingly common among hospitals, often classified as quality improvement or under the umbrella of antimicrobial stewardship. Examples include targeted staff education with regard to test ordering, interpretation, or proper specimen collection, as well as laboratory “prior authorization” policies designed to limit tests. In the near future, the CMS may begin to require diagnostic stewardship in the form of an approved clinical-decision support system, to receive full payment for advanced diagnostic imaging tests (through the Appropriate Use Criteria program established under the Protecting Access to Medicare Act of 2014, pending final approval by the CMS).^5^

Diagnostic stewardship has a potentially important role in HAI surveillance. The Centers for Disease Control and Prevention (CDC), through the National Healthcare Safety Network (NHSN), monitors >70% of all US hospitals for several hospital-related infections including SSI, CLABSI, CAUTI, ventilator-associated pneumonia (VAP) (now more broadly characterized as a possible ventilator-associated pneumonia, or PVAP), and healthcare-facility-onset *Clostridium difficile* infection (HO CDI).^4^ Surveillance-based definitions, such as those developed by the NHSN for HAI events, are pragmatically designed for surveillance purposes and are not intended for use in the clinical evaluation and care of patients. For example, current NHSN surveillance definitions for HO CDI require only a positive test for *C. difficile* from an unformed stool specimen on or after hospital day 4, irrespective of patient symptoms, clinical condition, alternative diagnoses, or multistep testing laboratory algorithms, whereas clinical practice guidelines require clinical indications of disease and advocate that testing of asymptomatic patients is not clinically useful.^6,7^ Many surveillance definitions cannot necessarily be used to distinguish true infections from false-positive tests.

Overuse of tests is predicted to increase false positives that trigger needless downstream cost and treatment that may cause harm for the patient. Conversely, test underuse risks missed diagnoses and potential harm related to untreated conditions. As with antimicrobial utilization, we hypothesize that there exists a state of optimal test use for HAIs in at-risk patients.

HAI rates based on surveillance definitions may over-diagnose CAUTI, CLABSI, HO CDI, hospital-acquired pneumonia (HAP), and VAP, estimated up to 37%,^8^ 30%,^9^ 15%–53%,^10,11^ 47%,^12^ and 58%–68%,^13,14^ respectively. Furthermore, the results of new, highly sensitive molecular diagnostics that detect minute amounts of a microbial target, such as nucleic acid amplification testing (NAAT) for *C. difficile* toxin gene(s), may identify colonized rather than clinically infected patients. This misattribution of colonized patients can artificially increase HAI rates.^11^

Diagnostic stewardship is defined as coordinated systems or user-based interventions designed to promote evidence-based utilization of diagnostic tests, with the primary goals of improving value and care quality and safely reducing cost. It has the potential to address falsely inflated HAI rates due to overtesting.^15,16^ Diagnostic stewardship has been described recently by Morgan et al^15^ to occur in three stages: preanalytic (test-related decision making and specimen collection), analytic (relating to laboratory practices including protocolized or reflex test algorithms), and postanalytic (eg, selective reporting of antimicrobial susceptibility data to encourage the use of narrower spectrum agents).

Diagnostic stewardship has been shown to effectively reduce a variety of unnecessary general inpatient medicine tests, from excessive or redundant daily inpatient labs to diagnostic imaging.^16,17^ Diagnostic stewardship strategies are varied and include user-based approaches (eg, auditing, price display, and provider feedback) and systems-based approaches (eg, modifications to the computerized physician order entry (CPOE) system requiring selection of an indication for testing and inappropriate specimen rejection).

## IMPLEMENTATION CHALLENGES AND SAFETY CONCERNS

While reducing unnecessary tests for HAIs can have many potential benefits for the patient and hospital, test underutilization raises the possibility for serious infections going undiagnosed and untreated. For example, while excessive *C. difficile* testing may identify patients with colonization or resolving infections (which is not only a waste of resources but also leads to unnecessary treatment), more restricted testing might result in unrecognized and untreated CDI (resulting in harm to individual patients and greater risk of cross infection) or empiric treatment for CDI without testing as a workaround (resulting in unnecessary treatment in a subset of patients). A major objective for diagnostic stewardship for HAIs is to identify the “sweet spot” of test utilization that minimizes overdiagnosis and false positive results while maximizing appropriately indicated testing and true positive results. This spot likely will be infection and population (eg, disease prevalence) specific.

Because HAI-related tests pose unique risks associated with reduced testing, which outcomes should be tracked to monitor patient safety? General outcome measures, as in ASPs, could include length of stay, antimicrobial resistance rates, antimicrobial use, CDI rates, mortality, and readmission. Potential comorbid complications tailored to the HAI(s) in question are also an essential stopgap that should prompt reconsideration for testing. For instance, following the introduction of a “stewardship of culturing” aimed at reducing CAUTIs, Mullin et al^18^ monitored overall rates of hospital-acquired (HABSI) infections, given the potential for complications of untreated urinary tract infection. However, outcome data in this and other HAI-related diagnostic stewardship studies were collected in aggregate and were not stratified to patients for whom the test was prevented and thus were at the highest risk for untreated infection. Ideally, prospective monitoring for HAIs should be performed for patients before and after diagnostic stewardship interventions to assess the direct patient-centered impact of these interventions in addition to aggregate data. These safety measures have largely been overlooked in the limited literature to date that has assessed diagnostic stewardship for HAIs, and incorporation in future studies presents significant logistical hurdles. Discordance between surveillance and clinical definitions for HAIs or those without a clear gold-standard clinical definition (eg, CDI) present challenges to evaluating safety when differentiating true positives remains elusive.

Similar to ASPs, there is no one-size-fits-all approach to reducing unnecessary HAI tests among all institutions. Information technology and CPOE capabilities, population characteristics, local ordering practices, HAI incidence, and laboratory test performance characteristics should all be taken into account when developing a diagnostic stewardship approach. Institutional factors, such as laboratory and stewardship activity, hospital administration support, and barriers such as provider pushback, are additional factors to consider. As with any quality improvement effort, process measures are also vital to ensure that stewardship interventions are having their intended effects, such as testing rates (including tests that are rejected from processing) and rates of the target HAI.

Table 1, incorporating the stages-of-testing concept of Morgan et al,^15^ lists examples of diagnostic stewardship strategies for HAIs from the literature as well as other potential strategies that could be used to optimize test utilization. As in ASPs, engineered flexibility is key in the event that special circumstances require deviation from prescribed practices, the diagnostic stewardship strategy fails to achieve intended goals, or patient harm is detected.

## CONCLUSIONS

Clinicians are faced with increasingly complex medical problems and varying test sensitivity and specificity that usually are not apparent to those ordering tests. Thus, understanding how to limit false positives without restricting appropriate testing has become a major challenge as well as an important opportunity for improving hospital infection control, infection prevention, and patient safety. As new diagnostic technologies proliferate, key metrics like clinical relevance and cost-effectiveness must be considered before such technologies are incorporated into clinical practice, and systems must be in place for stewardship of each new test before it is introduced into clinical practice.^32^

Established testing recommendations (preferably from professional societies or governing medical bodies) are essential to developing a stewardship strategy; however specific, useful consensus guidelines for diagnostic testing for HAIs are often lacking. For instance, no clear consensus exists to guide the use of repeated blood culturing to minimize false-positive rates and maximize true positives, as in patients with repeated fevers and/or patients who are already on antibiotics.^33^

Developing meaningful guidelines for diagnostic stewardship for HAIs requires quality evidence from thoughtfully conducted clinical studies. Much work remains to be done to determine the safety and efficacy of limiting providers’ autonomy for HAI-related diagnostics. Outcomes and safety-oriented quality improvement research may help bridge the gap between clinical research and practice.

A combined diagnostic and antimicrobial stewardship model could promote better patient evaluations, test choices, interpretations of results, and decisions to prescribe antimicrobial therapy.^32^ Expanding on the success of antimicrobial stewardship, diagnostic stewardship should take a multidisciplinary, collaborative approach to existing best practices for HAI prevention.

## Figures and Tables

**TABLE 1.** Examples of HAI-related Diagnostic Stewardship Strategies

			Diagnostic Stewardship Strategies		
HAI	Guidelines	Guidance to Support Stewardship Approach	Preanalytic	Analytic	Postanalytic
CAUTI	ACCCM/ IDSA guidelines for evaluation of new fever in critically ill patients^19^	Urine culture should only be obtained in febrile catheterized patients when urinary tract is suspected as a source or if urinary obstruction, neutropenia, or recent surgery is present. Urine dipstick is not recommended for catheterized patients.	Multifaceted approach in an ICU setting including “stewardship of culturing,” reduced CAUTI rates by a third.^18^ BPA discouraging dipsticks for catheterized patients.	Reflex urine culture protocol instituted for immunocompetent ICU patients associated with lower CAUTI rates. The lab performed urine culture only if pyuria was present on urinalysis.^20^	Clear interpretative language (eg, “likely contaminant”) attached to result.
CDI	AAP guidelines for CDI in infants and children^21^ IDSA/SHEA guidelines for CDI in adults^7^	Avoid *C. difficile* testing in <1-year-olds and consider testing in children 1–2 years old only after alternative diagnoses are sought. Testing only for symptomatic patients with diarrhea and suspicion for CDI (accounting for patient risk factors, eg, recent antibiotics).	Clinical decision support tools effectively reduce inappropriate *C. difficile* tests in pediatric patients (<3 years old)^22^ and adults.^23^ Lab refusal of inappropriate (eg, formed) specimens significantly reduces tests.^24^	A 2-test algorithm (“screening” immunoassays for GDH and *C. difficile* toxins A/B followed by “confirmatory” NAAT) was a cost-effective approach to *C. difficile* testing at one medical center.^25^	Text accompanying negative NAAT results with explanation of high negative predictive value and discouraging retesting shortly afterwards unless clinical condition changes.
HABSI/CLABSI	IDSA clinical practice guidelines for intravascular catheter-related infection^6^	Blood cultures should be obtained by a specialized phlebotomist. Catheter-drawn cultures to be done only when catheter-related BSI is suspected, along with a peripheral sample. Meta-analysis shows catheter-obtained specimens more likely to be contaminated versus venipuncture.^26^	Policy discouraging routine blood culture samples drawn from central lines plus reeducation of phlebotomists reduced blood culture contamination and CLABSIs related to contamination.	Use of molecular microarray for gram-positive blood cultures shortens time to pathogen identification and appropriate antimicrobial therapy for patients with VRE bacteremia.^27^	Rapid microarray results coupled with mandatory infectious diseases consultation for positive gram-positive cultures reduced mortality due to *S. aureus* bacteremia.^28^
VAP	ATS/IDSA guidelines for management of hospital-acquired and VAP^29^	Empiric antimicrobial therapy based on local antibiogram, with noninvasive specimen sampling (with semiquantitative culture) are recommended for suspected VAP. “Surveillance” respiratory specimens are not recommended and prospective evidence support this approach.^30^	Provider education, test auditing, and/or feedback regarding appropriate noninvasive sampling strategies for management of VAP.	Rapid molecular testing for MRSA in lower respiratory specimens for VAP may facilitate earlier antibiotic de-escalation.^31^	Microbiology results coupled with recommended VAP diagnostic thresholds (CFU/mL) for various sample types (eg, endotracheal aspirate vs BAL) and relative clinical utility of each type.

note. CAUTI, catheter-associated urinary tract infection; BPA, best practice alert; ACCCM, American College of Critical Care Medicine; CDI, *Clostridium difficile* infection; AAP, American Academy of Pediatrics; IDSA, Infectious Diseases Society of America; SHEA, Society for Healthcare Epidemiology of America; GDH, glutamate dehydrogenase; NAAT, nucleic acid amplification test; HABSI, hospital-acquired bloodstream infection; CLABSI, central-line associated bloodstream infection; VRE, vancomycin-resistant *Enterococcus*; VAP, ventilator-associated pneumonia; ATS, American Thoracic Society; MRSA, methicillin-resistant *Staphylococcus aureus*; CFU, colony-forming units; BAL, bronchoalveolar lavage.
